# UV Photoprotection, Cytotoxicity and Immunology Capacity of Red Algae Extracts

**DOI:** 10.3390/molecules24020341

**Published:** 2019-01-18

**Authors:** Félix Álvarez-Gómez, Nathalie Korbee, Virginia Casas-Arrojo, Roberto T. Abdala-Díaz, Félix L. Figueroa

**Affiliations:** Department of Ecology, Faculty of Sciences, Campus Universitario de Teatinos s/n, Malaga University, 29071 Malaga, Spain; nkorbee@uma.es (N.K.); virginiac@uma.es (V.C.-A.); abdala@uma.es (R.T.A.-D.); felix_lopez@uma.es (F.L.F.)

**Keywords:** cytotoxicity, cytokine production, interleukin-6, mycosporine-like amino acids, photoprotection, red algae extracts, tumor necrosis factor-α

## Abstract

This study was designed to evaluate the potential use of algal extracts in cosmeceuticals, including factors related to biosecurity. The aqueous crude extracts of *Hydropuntia cornea* and *Gracilariopsis longissima* showed a good photoprotective capacity (Sun Protection Factor, SPF) due to, among other reasons, the presence of five types of mycosporine-like amino acids (MAAs) detected by high pressure liquid chromatography-photodiode array detector (HPLC-PDA) and electrospray ionization mass spectrometry (ESI-MS) (Palythine, Asterina-330, Shinorine, Porphyra-334, and Palythinol). The toxicity of the extracts was evaluated by the MTT assay, which is based on the metabolic reduction of MTT [3-(4,5-dimethylthiazol-2yl)-diphenyl tetrazolium bromide] by the action of the mitochondrial enzyme succinate dehydrogenase. This assay was carried out in vitro in three cell lines: one related to the immune system (murine macrophages of the immune system: RAW264.7) and two human cell lines related to the skin (gingival fibroblasts: HGF, and immortalized human keratinocytes: HaCaT). Both extracts showed no cytotoxic activity in both types of human cells, whereas they showed cytotoxicity in murine tumor cells of the immune system (macrophages: RAW264.7). On the other hand, the immunological activity in the murine macrophage RAW264.7 was studied at a concentration lower than 100 μg mL^−1^ and lower than the EC_50_, and evaluated by the production of pro-inflammatory compounds through an immunosorbent assay linked to enzymes such as tumor necrosis factor-α (TNF-α) or anti-inflammatory/proinflammatory enzymes such as interleukin-6 (IL-6). Both algae extracts induced the biosynthesis of TNF-α and IL-6. The production of TNF-α was much higher than that observed in the control (at a concentration of the aqueous extract higher than 5 μg mL^−1^). These results support the theory that the extracts of *H. cornea* and *G. longissima* actively induce the production of cytokines. In summary, the extracts of these species did not show cytotoxicity in human cells, and they present with immunomodulatory and photoprotection capacity.

## 1. Introduction

The cytotoxic effects of algal extracts on different cell lines are currently being studied largely due to the safety requirements of the cosmetic industry [1,2]. Within these studies, cytotoxic activity in cancer and tumor cell lines has become one of the most important specificities of algae; in fact, many species have shown such bioactive effects [3,4]. Additionally, the dermocosmetic industry is investigating the application of new components in their products that have cytotoxic effects in melanoma and other skin disorders [5], but without cytotoxic activity in dermal cells [6]. The algal bioactive compounds, both in crude extracts and once isolated, can have a cytotoxic effect on cancer and tumor cells, as well as immunomodulatory activity [7,8,9,10]. The research in this field also focuses on elucidating which specific compounds demonstrate such bioactivities, although the synergies that occur in algal extracts have also attracted the interest of the scientific community, since this supposes a considerable increase of the bioactive capacity due to the interactions that occur between substances [11].

Regarding the immunological properties of the compounds obtained from algae, the studies that have been carried out aimed to induce macrophage secretions of cytokines (interleukin IL-6) and activation of tumor necrosis factors (TNF-α), as well as mediators of inflammation such as nitric oxide (NO), among others [8]. The main role of TNF-α is the regulation of immune cells, and in addition it can act to reduce viral replication [12]. This cytokine is involved in systemic inflammation and stimulates the acute phase reaction. On the other hand, IL-6 is a multifunctional cytokine that can be proinflammatory or anti-inflammatory depending on the cell lines regulating several immune responses, including acute phase reactions, and it participates as a mediator of the inflammatory response [13]. Its production is induced by several factors, including TNF-α, IL-1β, and bacterial endotoxin lipopolysaccharides [14].

Within the molecular diversity present in algae, polysaccharides are perhaps the most interesting components in terms of immunological effects. In particular, sulfated polysaccharides can trigger cellular or humoral stimulation of the human immune system [15]. Effective fractions of polysaccharides were found mainly in cyanobacteria, although recently, the potential of red and green algae compounds as potent immunomodulatory agents has been shown [16,17]. On the other hand UV photoprotectors, mycosporine-like amino acids (MAAs) extracted mainly from red algae [18,19], also present with immunological activity [20,21].

In this study, the cytotoxicity of an algal extract from two red algae, *Hydropuntia cornea* and *Gracilariopsis longissimi*, is evaluated. These two species are of interest in cosmeceutical applications due to their high antioxidant and photoprotection capacity related to MAAs and phenolic compounds [18,19], as well as their immunological activity. *Hydropuntia cornea* polysaccharides have been shown to have antiviral and antiparasitic effects [22,23], and the extracts of *Gracilariopsis longissima* have been shown to have antimicrobial properties [24,25], as well as an antidiabetic molecule arising from its lipid composition [26].

## 2. Results

### 2.1. Mycosporine-Like Amino Acids (MAAs)

In addition to HPLC, the UV-absorbing/screening compounds were characterized by ESI-MS. The ESI-MS analysis of MAA displayed a prominent ion peak of protonated molecules ([M + H]^+^) at *m*/*z*, which is consistent with the mass spectrometry analysis of MAAs derived from algae [27]. Based on their on-line UV-visible absorption spectra and mass spectrometry, MAAs were identified in both algae as Palythine (UV λ_max_ 320 nm, *m*/*z* 245.11290 in *H. cornea* and 245.11290 in *G. longissima*), Asterina-330 (UV λ_max_ 331 nm, *m*/*z* 289.13889 and 289.13895), Shinorine (UV λ_max_ 334 nm, *m*/*z* 333.12878 and 333.12863), Porphyra-334 (UV λ_max_ 334 nm, *m*/*z* 347.14462 and 347.14435) and Palythinol (UV λ_max_ 334 nm, *m*/*z* 303.15479 and 303.15460) for *Hydropuntia cornea* and *Gracilariopsis longissima*, respectively (Table 1). In both species, all MAAs found were detected with a level of accuracy of less than 2 ppm.

The main MAA in *H. cornea* in terms of percentages with respect to the total amount of MAAs was Palythinol (49.2%), and in *G. longissima* was Asterina-330 (42.9%). The total MAA content per gram of dry weight of biomass in *G. longissima* was two times higher (1.6 mg MAAs g^−1^ DW) than that in *H. cornea* (0.8 mg MAAs g^−1^ DW) (Table 1).

### 2.2. Sun Protection Factor (SPF)

**Figure 1:**
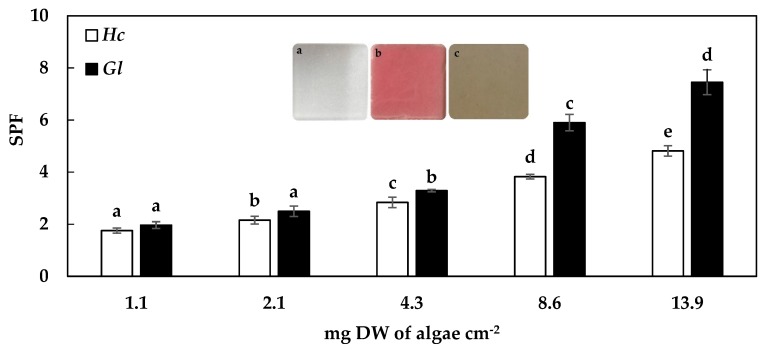
Photoprotection values (SPF, 290–400 nm) for the extracts of *Hydropuntia cornea* (*Hc*) and *Gracilariopsis longissima* (*Gl*) at different concentrations. The letters on the histogram bars (**a**–**e**) correspond to significant differences between the concentration for each species studied in a post-hoc analysis (SNK) for a one-way ANOVA. The three images in the middle correspond to the plate PMMA without extract (**a**), with *Hydropuntia cornea* (**b**), and with *Gracilariopsis longissima* (**c**) extracts at 8.6 mg DW cm^−2^.

Regarding the photoprotective capacity of the algae extracts of *H. cornea* and *G. longissima*, an exponential increase of the SPF values related to the increase of extract concentration was observed (*p* < 0.05, R^2^ = 0.99 and 0.97, respectively). At low concentrations in the PMMA plate (1.1 to 4.3 mg DW of algae per cm^−2^), *H. cornea* and *G. longissima* showed slight differences in SPF values. However, at higher concentrations (up 4.3 mg DW of algae per cm^−2^), the results differed between species. The highest values of SPF found in this study were 7.5 for *G. longissima* and 4.8 for *H. cornea*, at 13.9 mg DW of algae per cm^−2^ (Figure 1).

### 2.3. Cytotoxicity Assays

The cell viability (expressed as EC_50_ in mg mL^−1^) was tested by incubating the cell lines in a MTT solution, and determined the degree of affectation caused by the algal extracts in these cell lines. Based on this assay, cell toxicity values expressed as EC_50_ in the macrophage cells (RAW264.7) after 72 h of incubation with the extracts for *G. longissima* and *H. cornea* extracts, respectively, are shown in Table 2.

The results on the cytotoxicity of the algal extracts against the human fibroblast cell line (HGF) through the MTT assay are shown in Table 2. Based on the data obtained, it was concluded that 4.2 mg mL^−1^ of *G. longissima* extract and 250.7 mg mL^−1^ of *H. cornea* extract showed significant effects on cell integrity (Table 2).

In the HaCaT cell line, no biological effects were observed in the cells incubated with *G. longissima* extract, while the *H. cornea* extract caused a reduction in cell viability of 50% in this cell line (Table 2).

### 2.4. Immunology Results

**Figure 2:**
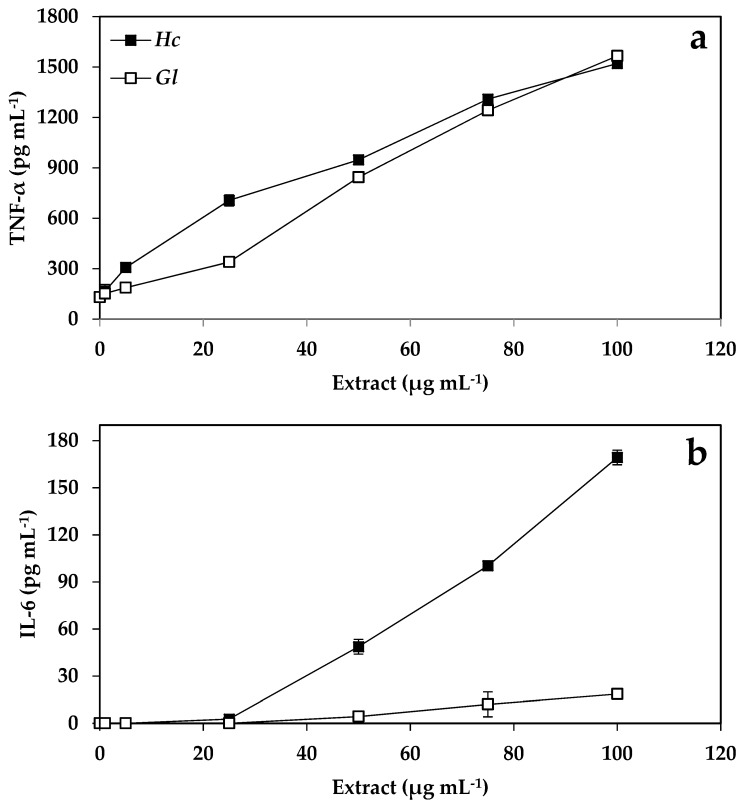
Concentration of tumor necrosis factor (TNF-α) synthesized by RAW macrophages (pg mL^−1^) (**a**) and production of interleukin-6 (IL-6 pg mL^−1^) by RAW macrophages (**b**) exposed to different concentrations of extracts of *H. cornea* and *G. longissima*. Data are expressed as the mean of three samples ± SE.

The synthesis and accumulation of cytokines increased as a response to the increase of the extract concentration of *H. cornea* and *G. longissima*, with 4 pg mL^−1^ the lowest concentration detected according to the sensitivity of the method. The control for LPS (bacterial lipopolysaccharide as an inducer of the immune system) in TNF-α was 23.8 ± 7.8, and in IL-6 was < 4 pg mL^−1^. An increase of 11.7 and 12 times more TNF-α was observed when the concentration of the extracts increased from 0 to 100 μg mL^−1^ in *G. longissima* and *H. cornea*, respectively (Figure 2A). The production of TNF-α was significantly higher than that of the control when the concentration of the extracts of both species was greater than 5 μg mL^−1^. Similar to the TNF-α trend, IL-6 levels increased linearly from 50 to 100 μg mL^−1^ in *G. longissima*, whereas in *H. cornea*, a linear increase was observed but with a very low slope with these same extract concentrations (Figure 2B).

## 3. Discussion

### 3.1. Mycosporine-Like Amino Acids (MAAs)

Algae have mechanisms to attenuate the effects generated by the UVR and, as such, they present with ecophysiological strategies that have already been widely studied [28]. These mechanisms basically consist of the biosynthesis of chemical compounds, induced by the UVR, that help to counteract deleterious effects—Such as MAAs and phenolic compounds [29,30,31]. Numerous studies have shown that MAAs prevent 3 out of 10 photons from hitting UVR-sensitive targets at the cytoplasmic level. Cells with high concentrations of MAA are 25% more resistant to UVR than those with low concentrations, or in organisms without MAAs [32]. The MAAs are secondary metabolites with multiple uses and, due to the powerful absorption effect, they protect the cells against UVR through both their antioxidant and photoprotection properties [33]. They have various cosmeceutical and pharmaceutical applications since, in addition to the above cited properties, they are highly thermostable and photostable molecules [34]. More than 35 different MAAs have been identified in marine organisms such as cyanobacteria, algae, and vertebrate and invertebrate animals [21]. MAAs are more abundant in Rhodophytes (as in the species in this study), compared to Chlorophytes and Phaeophytes [35,36].

### 3.2. Sun Protection Factor (SPF)

The current market in cosmetics has increased during the last decade [37]. In recent years, most cosmetics companies have launched cosmeceutical products which contain sun photoprotectors, moisturizers, antioxidants, or a combination of the three, and also provide bioactive ingredients capable of improving cellular functions related to antioxidant defence [38,39]. The high absorption of the algal extracts of *H. cornea* and *G. longissima* should be associated with their content in photoprotective molecules: total MAAs (0.8 and 1.6 mg MAAs g^−1^ DW), the content of phycobiliproteins (phycocyanins + phycoerythrins) which give the extract its “pink color” (up to 1.2 mg PC g^−1^ DW and 5.3 mg PE g^−1^ DW), and the phenolic content (up to 95 mg of phenolic compounds g^−1^ DW in both species) [40].

*H. cornea* and *G. longissima* have MAAs which act as biological photoprotectors, absorbing UVA and UVB radiation [35]. According to previous research in the field, Porphyra-334 (detected in both *H. cornea* and *G. longissima*) is one of the MAAs with the highest potential to be used as a photoprotector in cosmetic products, due to its photophysical and photochemical properties [41,42]. Currently, there are commercial cosmetic products on the market that include Porphyra-334 (Helioguard 365^®^). In vivo tests have been carried out using a cream with 5% Helioguard 365^®^ (final MAA concentration of 0.005%) and the same base with 4% of a synthetic UVB sunscreen and 1% of a synthetic UVA sunscreen. Helioguard 365^®^ improved the firmness and smoothness of the skin, as well as wrinkles in the area of application of the product after 4 weeks of use [43]. Helionori^®^ is another product that offers natural photoprotection against sunburn and contains, as bioactive ingredients, three MAAs: Palythine, Porphyra-334 and Shinorine, extracted from the red algae *Porphyra umbilicalis*. The formulation is resistant to sun exposure for 6 h and at 120 °C, and has a stability of 18 months when stored at a temperature of 15–25 °C. The application of a cream with 5% Helionori^®^ effectively prevented the appearance of burns by 94% compared to a control [44]. In addition, the formulation exhibited a notable protective effect on the metabolism of fibroblasts and keratinocytes exposed to oxidative stress induced by UVA. After 24 h of irradiation in the presence of 2% Helionori^®^, the protection of the keratinocytes increased by 57%, and the fibroblasts increased by 135%. The product also provided protection of the cellular components against UVA. The application of 2% of Helionori^®^ conserved membrane lipids of keratinocytes by 139% and fibroblasts by 134%, and also offered maximum protection for DNA [44]. The red algae tested in this study, due to the content of MAAs and its antioxidant capacity [18,19], is interesting for its potential use in the cosmetic industry. Asterina-330, the major MAA in *G. longissima*, has been reported to have a high capacity to protect against lipid peroxidation according to -carotene oxidation assay [45], whereas Shinorine from *G. longisssima* also has high antioxidant activity [45]. This MAA has been reported, together with Porphyra-334, to have a high protection capacity against UV-induced skin damage in mice, preventing sun burn cell formation and other morphological alterations, reducing the level of heat shock proteins, and maintaining the level of superoxide dismutase and catalase activities in the UV protected mice [46].

### 3.3. Cell Viability

The reduction of MTT depends on NADPH-dependent mitochondrial oxidoreductase enzymes [47,48], which are indicative of the physiological state. Therefore, the amount of living cells is proportional to the amount of formazan (metabolite of MTT degradation) produced [49]. In this study, the extracts of *H. cornea* and *G. longissima* had a cytotoxic effect at low concentrations on the RAW264.7 cells measured by the MTT assay. These algal species also have a high content of several halogenated phenolic compounds [50], which could have a cytotoxic capacity in some cell lines and in addition could have antioxidant, antimicrobial, anticancer, and antidiabetic capacities [51]. MAAs have protective effects against UVB-induced apoptosis and DNA fragmentation through the modulation of caspases [52].

In a study in which aqueous extracts of *Gracilaria corticata* were used for its potential antitumoral activity in the Jurkat and molt-4 cell lines (human leukemic cell lines), a good level of activity against the replication of the tumor cells was observed [53]. The most effective concentration used in the Jurkat and molt-4 cells was 9.3 and 9.7 mg mL^−1^, respectively, so the researchers concluded that the extract of this species did not show a significant cytotoxic effect in the molt-4 cells. However, its effect was demonstrated to be cytostatic in terms of inhibition against the development and multiplication of tumor cells [53]. The number of senescent cells related to Jurkat cells was higher than that of molt-4 cells [53].

With respect to the cell viability observed in human gingival fibroblasts (HGFs), differences were observed between the cytotoxic effects originated by the extracts. *G. longissima* extract has a cytotoxic effect about 59 times greater than that of *H. cornea* extract as far as EC_50_ values are concerned. Thus, cell viability and toxicity in cell lines related to the dermis are critical criteria for the evaluation of extracts obtained from these species.

A study conducted using the short-term toxicity test recommended by the International Organization for Standardization (ISO) (ISO 10993-12) [54] revealed that MAAs such as Shinorine, Porphyra-334 and Mycosporine-Glycine are not toxic to murine fibroblasts. This was confirmed in a second assay of direct longer-term incubation in the same cell line. After 14 and 21 days of incubation with the different MAAs, there was no significant toxicity and only minor effects on cellular morphology were observed for some of the MAAs [55]. Another study showed that the same three MAAs were non-toxic in human lung fibroblast cells (TIG-114) at concentrations between 0–100 μM after 48 h, and on the contrary they actually increased cell proliferation [56]. These findings were confirmed by Kim et al. (2014) [57] when studying the viability of the same MAAs. Porphyra-334 has also been shown to have no effect on the cell viability of human skin fibroblasts at concentrations up to 200 μM [58]. With respect to HaCaT, none of the extracts showed toxicity to this cell line. Only in *H. cornea*, at very high concentrations, was a reduction in cell viability observed (expressed as EC_50_ (259.5 mg mL^−1^)). In contrast, *G. longissima* showed an increase in cell viability, even beyond 100%. Similar results were found by studying the MAAs Shinorine, Porphyra-334 and Mycosporine-Glycine which, in isolation, significantly reduced cell viability in HaCaT keratinocytes in different proportions at concentrations of 0.1 mg mL^−1^ and above [59]. On the other hand, Fernandes et al. (2015) [55] found that Mycosporine-Glycine immobilized in a biofilm of chitosan induces cell proliferation (murine fibroblasts L-929). Therefore, the MAAs present in *H. cornea* and *G. longissima* (0.8 and 1.62 mg MAAs total g^−1^ DW, respectively) [40] could have effects on cell proliferation, as reported by Choi et al. (2015) [59]. There are also several studies that demonstrate that MAAs prevent UVR-induced toxicity. This protective effect has also been demonstrated in colemin A (a compound with a chemical structure related to MAA), where the UVB exposure of HaCaT keratinocytes through a quartz plate coated with this metabolite produced an increase in cell viability, demonstrating a proliferative effect [60].

In another in vitro experiment where fibroblasts were exposed to UVA radiation, the application of Porphyra-334 at concentrations of 10–40 μM also prevented the reduction of cell viability and the induction of senescence [58]. Therefore, the application of this MAA after exposure to UVR contributes to greater cellular viability compared to the control, and these results would support the use of this metabolite in post-solar creams. Suh et al. [52] found that the proliferation rate of HaCaT cells irradiated with UVR is significantly reduced (by up to three times) compared to unirradiated cells. Under UVR exposure, HaCaT cells showed an increase in cell viability after treatment with Porphyra-334. In particular, pretreatment with Porphyra-334 attenuated the inhibitory effects of UVR, resulting in high cell survival rates in HaCaT (up to 88%) compared to unirradiated cells. This study suggests that Porphyra-334 would contribute to the mitigation of UVR-induced apoptosis and DNA fragmentation. To determine if Porphyra-334 affected the metabolic pathway of caspases in HaCaT cells, caspase-3 protein levels were measured. After irradiation of HaCaT cells, activation of caspase-3 (based on decreased expression of procaspase-3) was clearly observed. The study revealed the attenuation of apoptotic signaling in cells treated with Porphyra-334. In particular, the levels of procaspase-3 and active caspase-3 in the cells treated with Porphyra-334 were restored to ~80% of the control levels, which implied that the protection of the cells against UVR damage by Porphyra-334 was partially mediated by the suppression of caspase activation [52].

### 3.4. Cytokine Production

This study shows that aqueous extracts of *G. longissima* and *H. cornea* stimulate the production of cytokines of both TNF-α and IL-6 in macrophages of the cell line RAW264.7. This activation may be due to the fact that both species contain chemical compounds with pro- and anti-inflammatory properties, attributed to their great diversity of metabolites of different natures [61]. Other studies have shown that seaweed extracts increase the phagocity and secretion activity of macrophages [62,63]. Extracts of the red algae *Porphyra yezoensis* have been shown to induce the production of TNF-α both in vitro and in vivo for murine macrophages [64]. The acid polysaccharides of *Halopithys incurva* induce the production of IL-6 and nitric oxide [8], while those of the red microalgae *Porphyridium cruentum* induce both TNF-α and IL-6 in macrophages of the cell line RAW264.7 [7]. Similarly, Yim et al. [65] demonstrated that the exopolysaccharide p-KG03—Containing high levels of sulfate produced by the dinoflagellate microalga *Gyrodinium impudicum* strain KG03—Increased the production of macrophage cytokines such as IL-1β and IL-6, as well as TNF-α. In this study, the extracts of *G. longissima* and *H. cornea* also induce the release of cytokines much more than that observed for the cytokine inducer LPS (bacterial lipopolysaccharide) used as a control—Up to 15 times more for TNF-α, and 2 times more for IL-6. Other studies with red algae of the family Gracilariaceae, specifically *Gracilaria verrucosa* (heterotypic of *Gracilariopsis longissima* for this study according to Steentoft et al. [66]), indicate that they are inhibitors of the production of proinflammatory mediators (NO, IL-6, and TNF-α) due to the bioactivity exerted by two fatty acids (C9 and C10) contained in their composition [67]. Another study carried out by Saeidnia et al. [26] revealed that cholesterol is one of the main sterols in the genus *Gracilariopsis,* as has been reported in the scientific literature for most red algae (Rhodophyta) [68]. The concentration of cholesterol, the typical sterol in red algae, is also important in brown algae. Fucosterol, abundant in brown algae, has also been isolated from *Gracilariopsis persica* [69]. This metabolite has been of interest due to its antidiabetic activity. The administration of fucosterol (orally) at 30 mg kg^−1^ in diabetic rats induced by streptozotocin was shown to induce a significant decrease in serum glucose concentrations. In addition, administration of fucosterol (300 mg kg^−1^) in diabetic rats induced by epinephrine inhibited the rise of blood glucose levels and glycogen degradation [70]. Therefore, fucosterol could be a principal antidiabetic principle in marine algae of the genus *Gracilariopsis* [26,71]. In addition, fucosterol has been reported to be an antioxidant sterol of seaweed which acts by increasing antioxidant enzymes [72]. It has been reported that the β-sitosterol compound reduces the symptoms of Benin Prostatic Hyperplasia (BPH), and also has utility as an anti-inflammatory agent [73,74].

In addition to those described above, MAAs that are considered natural sunscreens (among many other roles) and are found in different species in moderate amounts can be exploited biotechnologically in various ways [40]. In *H. cornea* and *G. longissima,* high levels and diverse compositions of MAAs have been found under UV radiation, along with a high content of inorganic nitrogen [40]. There is evidence that MAAs (Shinorine, Porphyra-334 and Mycosporine-Glycine) protect human fibroblasts from UVR, as well as protecting cells from UVR-induced cell death [56]. Studies of the antiproliferative activities of MAAs extracted from marine red macroalgae suggest that MAAs have relevant pharmaceutical bioactivities in the human body. The immunomodulatory effects of the MAAs Shinorine and Porphyra-334 obtained from *Gelidium* sp. and *Ceramium* sp. have been investigated in human cell lines, showing the activation of anti-inflammatory pathways [21]. MAAs have increasing potential applications in cosmetics, as well as in UVR photoprotectors (oral and topical), and are now also being proposed as activators of cell stimulation [34,75].

In addition, the phenolic compounds present in *G. longissima* and *H. cornea* could be involved both in the total photoprotective capacity of the extract and in its anti-inflammatory properties [51,61,76]. Álvarez-Gómez et al. (unpublished) found bromophenolic compounds with anticancer properties [51] in *G. longissima* through ESI-MS with an observed atomic mass of 334.91954 and a calculated atomic mass of 334.91959 (ppm 0.1).

Currently, in the market, there are already preparations of high molecular weight polysaccharides, isolated from food grade microalgae, for use as immune system enhancers (for example “Immulin” from Spirulina platensis, “Immunon” from *Aphanizomenon flos-aquae,* and “Immurella” from *Chlorella pyrenoidosa* [77]). It has been shown that each of these polysaccharides substantially increase the levels of IL-1β and TNF-α, and are 10 times more active for in vitro activation of macrophages than preparations that are currently used clinically as immunotherapeutics. According to the results of the present work, the increase in the concentrations of TNF-α caused by *G. longissima* and *H. cornea* (1560 μg mL^−1^ of TNF-α in both species) are much higher that the induction caused by *Spirulina platensis* (30 μg mL^−1^ of TNF-α) found by Parages et al. [78].

Isolated MAAs such as Shinorine and Porphyra-334 stimulated NF-κB activity in unstimulated THP-1 blue cells in a dose dependent manner, with a more pronounced effect observed for Shinorine [21]. While Shinorine slightly superinduced NF-κB in LPS-stimulated cells, Porphra-334 reduced NF-κB activity in this inflammatory background [21]. These inflammatory pathways are affected by MAAs, but diverse effects were found depending on the specific MAAs [21].

The results of the present work indicate that the effect of aqueous extracts of *H. cornea* and *G. longissima* on RAW macrophages may be due to the different fractions present in the extract. However, more studies are needed to test this hypothesis, as well as experimentation with isolated metabolites that would demonstrate, individually, their biological effect on the production of cytokines in cells of the immune system.

## 4. Materials and Methods

The extracts of *H. cornea* and *G. longissima* were obtained by extraction from a lyophilized biomass with an aqueous solvent (Mili-Q^®^ water, Millipore Corporation, Burlington, Massachusetts, United States), according to the methodology described in Álvarez-Gómez et al. [18]. From these lyophilized extracts, 20 mg was weighed and dissolved in DMEN culture medium [8]. Subsequently, serial dilutions were made (up to a dilution of 1:512) in order to study the effects of the concentration of algal extracts on cell viability using the MTT assay with three cell lines: RAW264.7, HGF, and HaCaT (ATCC, Manassas, WV, USA). To study the immunomodulatory capacity of the extracts, immunoassays were performed for the cytokines TNF-α and IL-6, using the same extracts of *H. cornea* and *G. longissima* but at a concentration of 0–100 μg mL^−1^.

### 4.1. Mycosporine-Like Amino Acid Analyses

#### 4.1.1. High Pressure Liquid Chromatography-Photodiode Array Detector (HPLC-PDA)

MAAs were extracted in 20% aqueous methanol (*v/v*) from samples of dried algae. The samples were analyzed with a Waters 600 HPLC system (Waters Chromatography, Barcelona, Spain), as was described by Korbee-Peinado et al. [29]. Quantification was undertaken using published extinction coefficients [79]. Results of the analysis are expressed as total amount in mg g^−1^ DW, and as the percentage with respect to the total amount.

#### 4.1.2. Electrospray Ionization-Mass Spectrometry (ESI-MS)

Mycosporine-like amino acids were also analyzed by mass spectrometry (ESI-MS) with a high-resolution mass spectrometer (model Orbitrap Q-Exactive, Thermo Fisher Scientific, Waltham, MA, USA) provided with an electrospray ionization-heated probe (HESI-II), at the Central Service for Research Support (SCAI, University of Málaga, Málaga, Spain). The samples were dissolved in 100% methanol.

### 4.2. Evaluation of the Sun Protection Factor (SPF)

The calculation of the FPS was made according to the European method Colipa 2011 [80]. Five different concentrations of extract per unit area (1.1–13.9 mg DW of *H. cornea* and *G. longissima* per cm^−2^) were applied on the plates. For each concentration of algal extract studied, three plates (replicas) were prepared. The algal extracts were evenly distributed on the surface of a polymethyl methacrylate (PMMA) plate with a fingertip covered with a nitrile glove. Then, the plates containing the extract were placed between the beam path of a solar simulator and a Sphere Optics SMS-500 spectroradiometer (Contoocook, NH, USA.). The solar simulator used was a Spectra-Physics Model 66902 fitted with a mercury-xenon lamp (lamp power 50–500 W). This instrument generates the light source according to the reference standard, which must be between 51.4–63.7 W m^−2^ for the total irradiance (290–400 nm).

### 4.3. Cytotoxicity Assays

The cell lines that were used for the 3-(4,5-dimethylthiazol-2yl)-diphenyl tetrazolium bromide (MTT) assay were cultured in a DMEM medium supplemented with 10% fetal bovine serum, 2 mM l-glutamine, 100 units mL^−1^ of penicillin sodium, 0.1 mg mL^−1^ of sulphated streptomycin, and 0.25 μg mL^−1^ of amphotericin B. The cells were cultured at 37 °C (for 72 h) with humidified air containing 5% CO_2_ atmosphere until they reached confluence (75%).

The procedure to carry out the cell viability assay required preparing a cell suspension of 6 × 10^4^ cells mL^−1^ in DMEN culture medium. Thus, for the performance of the assay, the wells should contain approximately 3000 cells. Therefore, in a 96-well microplate the following volumes were added in quadruplicate: for the control 50 μL of DMEN medium + 50 μL of cell suspension medium; for the treatments 50 μL of extract (from an extract containing 20 mg of lyophilized algal extract in 1 mL of DMEN) + 50 μL of cell suspension medium. These were subsequently incubated for 72 h at 37 °C in a humid atmosphere and 5% CO_2_. After this time, 10 μL of MTT solution (5 mg MTT mL^−1^ of phosphate buffer solution) was added and incubated for 4 h. The crystalline precipitate that formed was solubilized with 150 μL of acid isopropanol (HCl 0.04 N). Finally, the optical density was determined at 550 nm in the microplate spectrophotometer (BIO-TEK, FL600, INC. Winooski, VT, USA). The results are expressed as a percentage (%) of living cells, according to the following equation:

Cell Viability (%) = (DO_treatments_/DO_control_) × 100 (1)

where DO_treatments_ is the absorbance of the treated cells, and DO_control_ is the absorbance of the control cells—That is, those that have not been subjected to the substance under study. For the calculation of parameter EC_50_ we used cell viability (%) versus concentration (mg extract mL^−1^).

### 4.4. Immunological Assays

For the determination of the cytokines, the cell line RAW264.7 was used as in the cytotoxicity assays. In this assay, RAW264.7 cells were cultured in the presence of different concentrations of algal extracts of *H. cornea* and *G. longissima* (0–100 μg mL^−1^) in 24-cell microplates (5 × 10^5^ cells well^−1^) in 1 mL total volume. Bacterial lipopolysaccharides (LPS) were used as a positive control (50 ng mL^−1^) for the activation of macrophages. The supernatant was collected after a 48 h incubation and used to determine cytokine production after stimulation by LPS. The production of TNF-α and IL-6 was determined by enzyme-linked immunosorbent assay (ELISA), according to the protocol described by Martínez et al. [14]. Subsequently, monoclonal rat anti-mouse TNF-α or IL-6 antibodies (0.5 mg, BD Pharmingen) were used to cover the cells at a concentration of 2 μg mL^−1^ at 4 °C for 16 h. After washing and blocking with saline PBS (phosphate buffer solution) containing 3% bovine serum albumin, the supernatants of the cultures were added to each of the cells for 12 h at 4 °C. The unbound material was washed and biotinylated anti-mouse monoclonal antibodies of TNF-α or IL-6 were added at a concentration of 2 μg mL^−1^ for 2 h. The fixed antibodies were detected by the addition of avidin peroxidase (Sigma-Aldrich, St. Louis, MO, USA) for 30 min, and the subsequent addition of an ABTS substrate solution. The absorbance at 405 nm was monitored after 10 min of substrate addition. A standard curve was constructed using various dilutions of recombinant murine TNF-α or IL-6 in PBS containing 10% FCS (fetal calf serum). The amount of each cytokine in the supernatants of the cultures was determined by extrapolation of the absorbances to the standard curve.

### 4.5. Statistical Analysis

Sun protection factor (SPF) was tested by an analysis of variance (ANOVA). Cochran´s test was used to check the homogeneity of variances. When the ANOVA results indicated significant differences, a post-hoc Student Newman-Keuls (SNK) multiple comparison test was applied. The level of significance (α) was set in all cases at 0.01.

## 5. Conclusions

The cytotoxicity caused by the extracts of *H. cornea* and *G. longissima* differs in terms of the cell lines used. Slight toxicity in macrophages when using both algal extracts and in gingival fibroblasts HGF in *G. longissima* extract was observed. The concentrations used in this study are high due to the methodological indications of previous tests, since we intended to investigate the range of concentrations at which the substance of interest is toxic or immunomodulatory. Although there is a lack of knowledge about the molecular mechanisms involved in the activation of macrophages by algae extracts, it has been suggested that this immune function could be similar to that observed in plant and mushroom metabolites studied in more detail than those from algae [81]. The results of this study suggest that aqueous extracts of *H. cornea* and *G. longissima* could be considered as potential nutraceutical, cosmetic and pharmacological products that would be applied when the activation of macrophages in inflammatory processes was necessary. These could stimulate the immune response of the cells by inducing the production of the cytokines TNF-α and IL-6, among others, in macrophages responsible for the immune responses. In addition to immunological activity, the extracts of both types of red algae show optimal photoprotection capacity due to the presence of MAAs, although other molecules such as phycoerythrin and polyphenols are not excluded as potentially contributing to this effect.

In this study, the effective concentrations are higher than other similar studies in which purified or isolated bioactive compounds were used instead of crude extracts [8,78]. Therefore, the fractionation and purification of *G. longissima* and *H. cornea* extracts in future studies are recommended. Even so, the use of algal extracts by the scientific community is of significant relevance due to the positive synergistic effects that occur among their components. This study proposes *G. longissima* and *H. cornea* as two very interesting red algae belonging to the family Gracilariaceae, with powerful bioactive effects tested in this and in other studies with several cell lines.

## Figures and Tables

**Table 1:** UV-sunscreen mycosporine-like amino acids (MAAs) isolated from *Hydropuntia cornea* (*Hc*) and *Gracilariopsis longissima* (*Gl*).

Species	MAA Type	%	Mol. Formula	λ_max_ (nm)	Exact (ppm)	Calculated (*m*/*z* [M + H]^+^)	Observed (*m*/*z* [M + H]^+^)
*Hc*	Palythine	29.9 ± 1.5	C_10_H_16_N_2_O_5_	320	1.2	245.11320	245.11290
	Asterina-330	12.9 ± 1.8	C_12_H_20_N_2_O_6_	330	1.8	289.13941	289.13889
	Shinorine	5 ± 1.5	C_13_H_20_N_2_O_8_	334	1.4	333.12924	333.12878
	Porphyra-334	3 ± 0.8	C_14_H_22_N_2_O_8_	334	0.8	347.14489	347.14462
	Palythinol	49.2 ± 3.6	C_13_H_22_N_2_O_6_	332	0.9	303.15506	303.15479
	Total MAAs	0.8 ± 0.1 mg g^−1^ DW					
*Gl*	Palythine	0.3 ± 0.1	C_10_H_16_N_2_O_5_	320	1.2	245.1132	245.11290
	Asterina-330	42.9 ± 1.1	C_12_H_20_N_2_O_6_	330	1.6	289.13941	289.13895
	Shinorine	41.2 ± 2	C_13_H_20_N_2_O_8_	334	1.8	333.12924	333.12863
	Porphyra-334	1.7 ± 0.1	C_14_H_22_N_2_O_8_	334	1.6	347.14489	347.14435
	Palythinol	13.9 ± 0.5	C_13_H_22_N_2_O_6_	332	1.5	303.15506	303.15460
	Total MAAs	1.6 ± 0.1 mg g^−1^ DW					

**Table 2:** Values of EC_50_ (mg mL^−1^) in cell lines of murine macrophage RAW264.7, HGF-gingival fibroblasts, and human HaCaT-keratinocyte for the species *Hydropuntia cornea* and *Gracilariopsis longissima*. The cells were treated with different extract concentrations (0–10 mg mL^−1^) for 72 h and evaluated by the MTT assay.

Species	RAW264.7	HGF	HaCaT
*Hydropuntia cornea*	0.12	250.7	259.5
*Gracilariopsis longissima*	0.41	4.2	-
